# Smartphone-Based Cardiac Rehabilitation Program Improves Functional Capacity in Coronary Heart Disease Patients: A Systematic Review and Meta-Analysis

**DOI:** 10.5334/gh.1253

**Published:** 2023-08-10

**Authors:** Bambang Dwiputra, Anwar Santoso, Budhi Setianto Purwowiyoto, Basuni Radi, Ade Meidian Ambari, Dwita Rian Desandri, Serlie Fatrin, Bashar Adi Wahyu Pandhita

**Affiliations:** 1 Department of Cardiology and Vascular Medicine, Faculty of Medicine, Universitas Indonesia/Harapan Kita National Cardiovascular Centre, Jakarta, Indonesia

**Keywords:** Coronary Disease, cardiac rehabilitation, smartphone, secondary prevention

## Abstract

Cardiac rehabilitation (CR) reduces mortality and morbidity in coronary heart disease (CHD); however, patients show a lack of adherence to CR. Alternatively, telehealth interventions have shown promising results for improving target outcomes in CR. This study aimed to review the effect of smartphone-based CR on the functional capacity of CHD patients. A literature search was performed using PubMed, MEDLINE, Embase, and Cochrane Library on 21 March, 2022 to find randomised controlled trials on smartphone usage in CR to improve functional capacity. Outcomes included maximal oxygen consumption (VO_2_ max), a 6-min walk test (6-MWT), quality of life, smoking cessation, and modifiable risk factors. Eleven trials recruiting CHD patients were reviewed. Wearable devices connected to smartphone- or chat-based applications were commonly used for CR delivery. Most trials managed to provide exercise prescriptions, education on medication adherence and controlling risk factors, and psychosocial counselling through the intervention. Functional capacity improved significantly following smartphone-based CR in CHD patients compared to control groups, as measured by VO_2_ max and 6-MWT; patients were more likely to quit smoking. Compared to traditional care, smartphones that delivered CR to CHD patients demonstrate superior outcomes regarding increasing functional capacity. There is no significant improvement on lipid profile, blood pressure, HbA1C, body mass index, and quality of life. It can be used either alone or as an adjunct. Ultimately, the patients’ preferences and circumstances should be considered.

## Introduction

Cardiovascular disease remains the leading cause of mortality globally, accounting for approximately 18.6 million deaths. Coronary heart disease (CHD) is the most prevalent type of cardiovascular disease. This number is expected to increase continuously with age in adults >20 years, owing to emerging epidemics of obesity, diabetes, metabolic syndromes, and an increasing aging population [1].

The European Society of Cardiology guidelines recommend primary percutaneous coronary intervention (PCI) as the preferred reperfusion strategy in acute coronary syndrome (ACS) patients because it has been proven to successfully lower mortality rates [2]. However, approximately 20% of survivors experience subsequent cardiovascular events, such as recurrent myocardial infarctions, strokes, or cardiovascular death; this creates significant economic and morbidity burdens. Therefore, the need for adequate long-term secondary prevention after ACS cannot be overstated [3].

Cardiac rehabilitation (CR) has been widely endorsed among several healthcare organisations owing to the growing body of evidence that has documented its positive outcomes, such as physiological improvement from exercise training, the psychological benefits of group support and counselling, better adherence to therapies, and control of cardiovascular risk factors [4]. Tobacco use, an unhealthy diet, obesity, physical inactivity, and the harmful use of alcohol are modifiable behavioural risk factors that put individuals at a higher risk of cardiovascular diseases [1]. Despite the benefits, program adherence is low. Common reasons include a lack of access to transport, ill health, and domestic or job responsibilities [5]. Especially during the COVID-19 pandemic era, movement was restricted to minimise the spread of infection. Healthcare providers are developing new innovations to deliver CR services based on patients’ own comfort via remote monitoring and tracking, smartphone-based online coaching, and virtual interviews. In Indonesia, home-based cardiac device use remains minimal [6]. Furthermore, traditional and community-based CR services have not been widely implemented in metropolitan areas, with even greater underrepresentation in rural areas. These reflect limiting barriers at the health service and system levels, which are significantly greater for people who live in rural settings, whereas the rates of CVD are similar in both urban and remote areas [7].

Therefore, information and communication technologies have been incorporated into healthcare delivery services to address patient-, health professional-, and system-related barriers. Innovative CR delivery includes various applications and platforms, allowing users to store and transmit data electronically. Smartphones have become ubiquitous in today’s population, with 92.1% of internet users using smartphones [8]. Smartphone-based interventions are accessible, scalable, and inexpensive. They are delivered via smartphone applications, websites, SMS messages, text messages, or video calls [9]. Several studies have outlined the efficacy, effectiveness, and acceptability of smartphone-based interventions in increasing engagement with healthcare teams and adherence to guideline-supported care plans, such as CR enrolment [5,6].

Despite its strong potential, evidence concerning the use of m-Health, particularly smartphones, in CR to improve functional capacity in CHD patients is emerging. There is considerable variation in study methodology, including the telehealth platforms used, and the overall value of this approach remains unclear. Therefore, this systematic review and meta-analysis aimed to compare the outcomes of smartphone-based CR to traditional CR/usual care for exercise tolerance in CHD patients.

## Methods

### Literature search strategy

A systematic literature search was performed using four databases: PubMed, MEDLINE, EMBASE, and the Cochrane Library. We also manually searched the bibliographies of relevant studies, systematic reviews, and conference proceedings.

The search date was 21 March, 2022. None of the included studies were limited by date or location. Studies not published in English were excluded. The search terms and strategies are listed in Table 1 of the Online Supplementary Material. (PROSPERO registration number: CRD42022325124).

### Study selection

Two independent reviewers (BD and SF) screened titles and abstracts to identify potentially relevant articles. Studies were considered randomised controlled trials investigating the effects of smartphone-based CR on functional capacity in CHD patients, compared with usual care or centre-based CR, with a follow-up period of at least 6 weeks. CR was defined as phase II or phase III programs with key intervention components, including exercise training, physical activity, advice on modification of cardiovascular risk factors, adherence to pharmacotherapy management, review of assessments, and psychosocial counselling. Generally, phase II CR is an early outpatient program delivering preventive and rehabilitative services that are performed within the first 6 months after a cardiac event, whereas phase III CR is a long-term program aimed at promoting the maintenance of healthy behaviours and CVD risk control [10]. Smartphone-based interventions can be delivered alone at patients’ own comfort or as an adjunct to centre-based CR. Methods of delivery included smartphone applications linked to wearable devices and social media chat platforms. Usual care involved routine clinical treatment and brief inpatient health education without ongoing rehabilitation support via smartphone interventions. Traditional CR is performed face-to-face in rehabilitation centres.

The included studies measured the outcomes of interest, such as maximal oxygen consumption (VO_2_ max), modifiable cardiovascular risk factors, quality of life, and smoking cessation rate. Study trials were excluded if the intervention was based mainly on the internet, telephone calls, or text messaging without using a smartphone. The study authors of trials that performed interventions in a population of CHD patients and other heart diseases were contacted to request a specific analysis of CHD patients only. Studies written in languages other than English were also excluded. Eligible studies underwent a full-text review by independent reviewers (BD and SF), and any disagreements on eligibility assessment or outcome data were resolved by discussion with other reviewers.

### Data extraction and analysis

Data were collected and recorded on a pre-developed data extraction form. Study design, location, patient demographics and baseline characteristics, methods of delivery, components of interventions, follow-up durations, and clinical outcomes were extracted. Outcomes included changes in functional capacity parameters, such as VO_2_ max, a 6-min walk test (6-MWT), and metabolic equivalent of task (MET). Other outcomes considered were changes in cardiovascular risk factors (total cholesterol, LDL-c, glycated haemoglobin [HbA1C]), quality of life, and smoking cessation rate. Continuous outcomes were transformed into uniform measurement scales, whenever necessary. MET were converted to oxygen consumption (1 MET = 3.50 mL/kg/min). Cholesterol levels were expressed in mmol/L (1 mg/dL = 0.02586 mmol/L). Authors were contacted to request information not available in the study reports. If the outcome assessments were performed at different time points, then the results with the longest follow-up period were included in the meta-analysis.

Critical appraisal of the randomised controlled trial was performed according to the guidelines of the Cochrane Handbook for Systematic Reviews of Interventions to assess the risk of bias in the selection, detection, attrition, and reporting of outcomes. The risk was judged as high, low, or unclear if the data were uncertain. Heterogeneity was evaluated qualitatively by comparing the study characteristics and quantitatively using I^2^ statistics. I^2^ values are categorised as low (25%), moderate (50%), or high (75%). A random effects meta-analysis was used if heterogeneity was identified, as indicated by I^2^ ≥ 50%. We performed a subgroup analysis when the heterogeneity was high.

Data synthesis and analyses were performed using Review Manager (V.5.4.1, Nordic Cochrane Centre, Copenhagen) in accordance with the Cochrane Handbook. Differences in the effects of smartphone-based and usual care/centre-based CR were also examined. The odds ratios (ORs) were used to assess dichotomous data. For continuous data, weighted mean differences and 95% confidence intervals (CIs) were calculated. Mean changes and standard deviations (SD) from the baseline values were used. In cases where these values were not reported, the SD was calculated from the available data using OpenMetaAnalyst [11]. Hypothesis testing was performed at a two-tailed 0.05 level.

## Results

### Study selection and characteristics

Preliminary searches of the four databases using the search terms yielded 152 studies. The keywords used for the search are summarised in Appendix 1. Three additional reports were identified by a manual search of the bibliographies of relevant studies or systematic reviews. After deduplication, 104 studies were screened based on their titles and abstracts. Eighty-six articles did not meet our eligibility criteria, and 18 full-text articles were retrieved. Five studies did not specifically include smartphones in their intervention groups. One study was not a randomised controlled trial. In another study, the required data were unavailable in the full paper, and attempts to contact the corresponding author were unsuccessful. Finally, 11 studies were included in this systematic review and meta-analysis. The study selection process is described according to the Preferred Reporting Items for Systematic Reviews and Meta-Analyses flow diagram in Appendix 2. The characteristics of the included studies are summarised in Appendix 3.

A total of 639 patients were included in the smartphone-based CR from 11 randomised controlled trials. The control group consisted of 646 patients. The baseline and clinical characteristics of the study participants are shown in Appendix 4. All trials enrolled patients diagnosed with CHD who had or had not received revascularisation therapy after ACS or had stable angina. One trial included patients other than those with CHD, such as those with heart failure, valvular heart disease, or arrhythmia [12]. Separate group analyses were requested by the author and rendered successful.

Smartphone-based CR was delivered via a smartphone application linked to wearable devices that tracked activity, heart rate, blood pressure, and ECG results in six trials. Four trials used smartphone applications only, in which patients could input their baseline medical data, tasks, and goals, and tailored motivational feedback was sent through the app. Two trials utilised social media chat platforms such as WeChat [13,14]. Among the 11 trials, three had incorporated routine telephone call monitoring in addition to the use of smartphone applications [14,15,16]. Components of interventions were similar among all trials, with physical activity or exercise training prescriptions, education on the modification of behavioural risk factors in cardiovascular diseases (smoking cessation and dietary habits), adherence to pharmacological treatments, psychosocial counselling, or individualised feedback on the performance of tracked activities. Two studies lacked healthcare team involvement in providing motivational feedback on implementing lifestyle changes [17,18]. Exercise prescriptions varied from 3–5 times per week, with a duration of 15 to 60 minutes of aerobic training, or a combination of aerobic and resistance training [19,20,21,22]. Intensity of exercise was recommended within a safe but effective heart rate zone or Borg scale of 11–13. To measure functional capacity, the intensity of maximum oxygen intake was evaluated in five trials [12,14,18,20,21]: 6-MWT in four trials [13,15,19,23], and peak MET in one trial [22].

Among the four phases of CR, only two studies focused on the third phase (the maintenance phase). The follow-up duration varied from 6 weeks to a year. This long-term review provided patients with continuous support for lifestyle changes, drug management, biopsychosocial well-being, and early interventions whenever necessary [12,19]. We concluded that other studies likely evaluated phase II CR in patients who were either recently diagnosed with CHD or had been discharged with a CHD diagnosis, as they were referred for secondary prevention programs. In two trials, smartphone-based CR was used as an adjunct to centre-based CR. The control group underwent standard care protocol or centre-based CR. The standard care protocol included routine follow-up assessments, ordinary medical therapy, brief inpatient health education, exercise encouragement at home, and self-management without tight supervision. Some studies have failed to elaborate on what their standard care protocols or control group interventions entailed. Centre-based CR was performed face-to-face under the supervision of a trained physiotherapist.

### Assessment of risk of bias

The quality of the trials was analysed using the Cochrane risk-of-bias tool for randomised trials (RoB 2). Participants in all trials were blinded to the allocation of treatments until they were recruited and assigned to the intervention; hence, bias due to the randomization process was low. In these studies, the participants could not have been blinded to the intervention because of its nature. However, trials by Lunde et al., Yudi MB, Dorje et al., Skobel et al., Maddison et al., and Widmer et al. managed to blind health outcome assessors to the primary outcomes [12,13,20,21,23]. Two trials provided no evidence of bias due to missing data [15,21]. Skobel et al. stated that the reasons for dropping out of studies were related to technical issues and minimal optimization of the safety threshold. Safety algorithms often hindered participants from fully completing the training programs, and patients were discouraged from participating until the specified follow-up period [21]. Because some studies used self-completed questionnaires to evaluate participants’ quality of life, knowledge of the intervention was likely to produce bias in the measurement of outcomes [12,17,19]. Overall, critical appraisal studies demonstrated low levels of bias for six studies, moderate bias for two studies, and high bias for three studies. Appendix 5 presents the results of the critical analysis.

## Effect of smartphone-based cardiac rehabilitation in CHD patients

### Functional capacity

**Figure 1:**
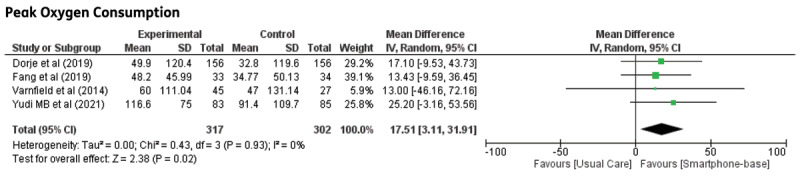
Maximal oxygen consumption in CHD patients undergoing smartphone-based cardiac rehabilitation (CR) and centre-based CR/usual care.

**VO_2_ max:** Five trials reported VO_2_ max as the outcome, and one trial [20] reported MET, which was converted to VO_2_ max (1 MET = 3.5 mL/kg/min^2^). All trials reported a favourable outcome in VO_2_ max, with statistically significant differences in studies by Lunde et al., Song et al., and Skobel et al. [12,14,21]. Meta-analysis results showed statistically significant improvement in VO_2_ max in groups receiving smartphone-based CR compared to control groups (MD: 1.48; 95% CI 0.54–2.41, p = 0.002). Although not significant, the trial by Escobar et al. demonstrated a contradictory result, probably because of the small number of participants and because all participants were male. This value contributed to the low level of heterogeneity in the pooled analysis. A forest plot of VO_2_ max is shown in Figure 1.

**Figure 2:**
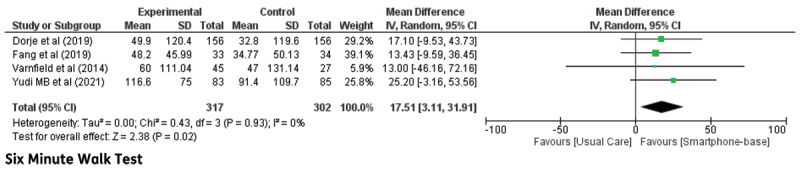
Six-minute walk test in CHD patients undergoing smartphone-based cardiac rehabilitation (CR) and centre-based CR/usual care.

**Six-minute walk test:** Four trials reported a 6-MWT. Meta-analysis of the included trials demonstrated significant differences between groups (MD: 17.51; 95% CI 3.11–31.91, p = 0.02). The short-term follow-up duration (6 weeks) in the study trial by Varnfield et al. did not demonstrate a significant effect in 6-MWT [19]. The forest plot of the 6-MWT is shown in Figure 2.

### Modifiable risk factors

**Figure 3:**
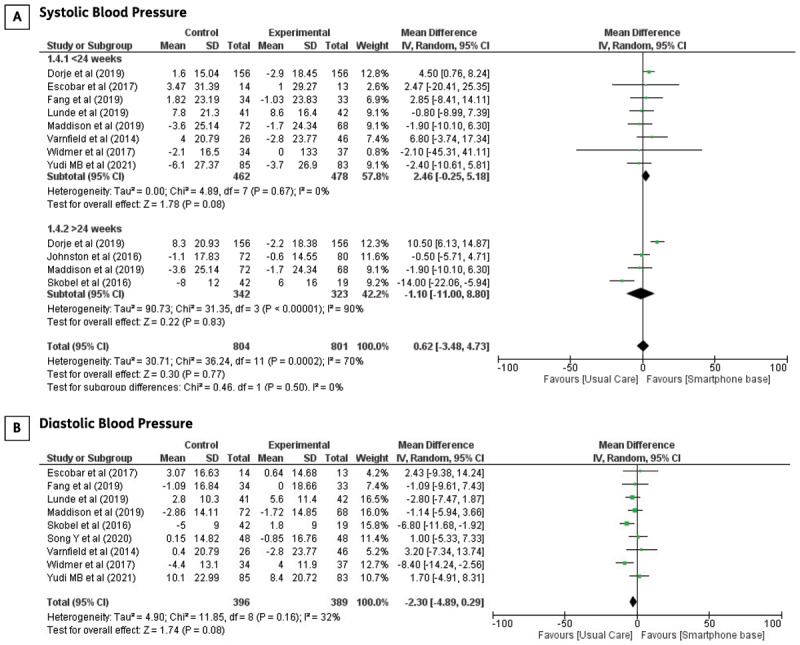
The effect of smartphone-based CR compared to centre-based CR/usual care in **A)** Systolic blood pressure **B)** Diastolic blood pressure in CHD patients.

**Systolic and diastolic blood pressures:** Nine studies evaluated the effects of smartphone-based CR on resting blood pressure. However, trials by Dorje et al. [13] and Johnston et al. [17] reported outcomes for systolic blood pressure alone, and the reason for this focus was not specified. Regarding systolic blood pressure, there was a high level of heterogeneity with non-significant results using a random-effects model. Therefore, subgroup analyses were conducted to determine the effects of the different follow-up periods. At the short-term follow-up (<24 weeks), systolic blood pressure did not significantly improve following smartphone-based CR compared to that in the control group (MD: 2.46; 95% CI –0.25–5.18, p = 0.08). Longer follow-ups did not yield significant results. There was evidence of significant heterogeneity in the long-term follow-up (I^2^ = 90%, χ_2_ = 31.35, df = 3, p < 0.00001). When the trials by Dorje et al. [13] and Skobel et al. [21] were removed from the meta-analysis, the heterogeneity was null, and the effect size remained insignificant. The meta-analysis of diastolic blood pressure was not statistically significant between the two groups (MD: –2.30; 95% CI –4.89–0.29, p = 0.08). The forest plots of systolic and diastolic blood pressure are shown in Figure 3.

**Figure 4:**
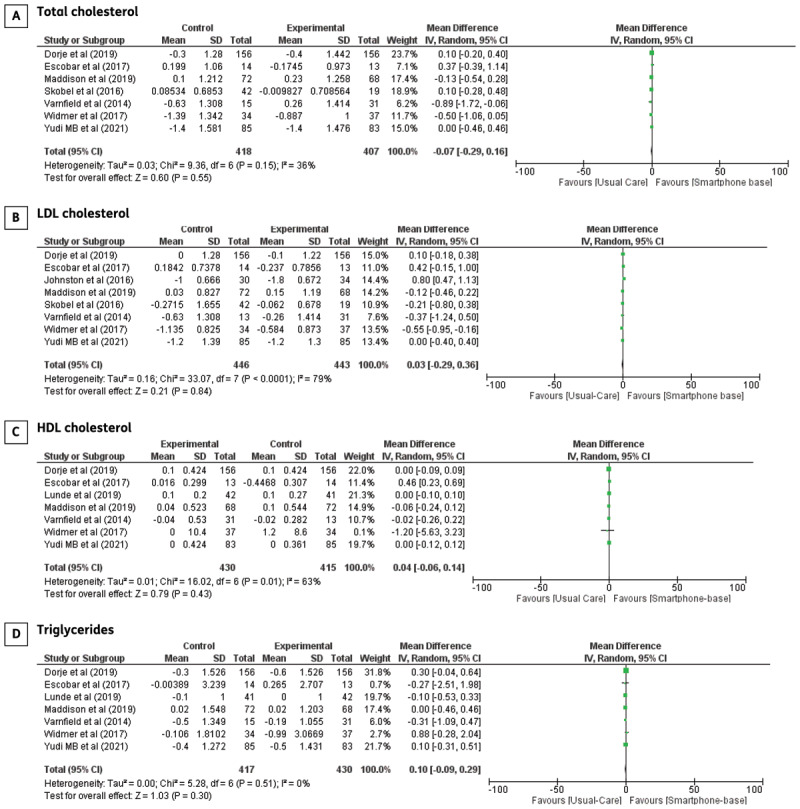
The effect of smartphone-based CR compared to centre-based CR/usual care in **A)** Total cholesterol **B)** LDL cholesterol **C)** HDL cholesterol **D)** Triglycerides in CHD patients.

**Blood lipids:** Eight trials investigated blood lipid parameters, including total cholesterol, LDL-c, HDL-c, and triglycerides in CHD patients undergoing smartphone-based CR, except for a trial by Johnston et al., which only reported results on LDL cholesterol. However, the reason for this finding remains unclear. No significant difference between smartphone-based CR and centre-based CR/usual care was found from meta-analyses of total cholesterol (MD: –0.07 95% CI –0.29–0.16, p = 0.55), LDL cholesterol (MD: 0.03 95% CI –0.29–0.36, p = 0.84), HDL cholesterol (MD: 0.04; 95% CI –0.06–0.14, p = 0.43), and triglycerides (MD:–0.10; 95% CI –0.28–0.09, p = 0.31). Forest plots of total cholesterol, LDL-c, HDL-c, and triglyceride levels are shown in Figure 4.

**Figure 5:**
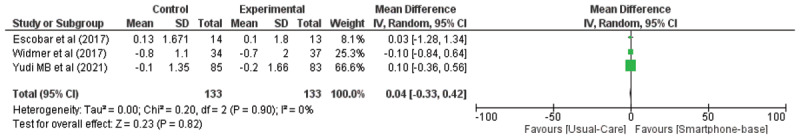
The effect of smartphone-based CR compared to centre-based CR/usual care in HbA1C in CHD patients.

**HbA1C:** Three studies compared HbA1c concentrations between smartphone-based and standard care/centre-based CR. No significant difference was observed (MD: 0.04; 95% CI –0.33–0.42, p = 0.82). Forest plots of HbA1C are illustrated in Figure 5.

**Figure 6:**
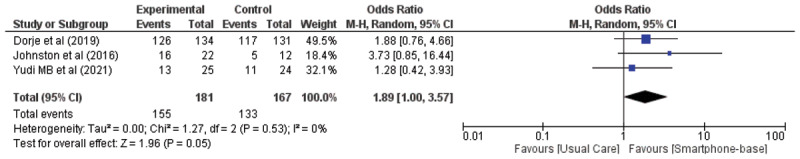
The effect of smartphone-based CR compared to centre-based CR/usual care in smoking cessation rate in CHD patients.

**Smoking cessation rate:** Prevalence of smoking cessation was found to be higher in groups that received smartphone-based CR compared to centre-based CR/usual care (OR: 1.89; 95% CI 1.00–3.57, p = 0.05). Forest plots of smoking cessation are shown in Figure 6.

**Figure 7:**
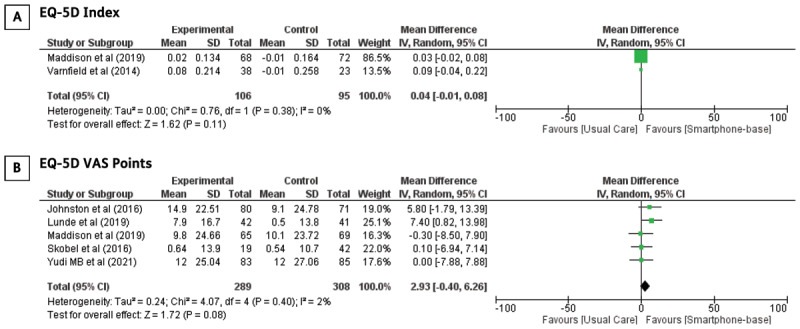
The effect of smartphone-based CR compared to centre-based CR/usual care in **A)** EQ-5D index and **B)** EQ-5D VAS in CHD patients.

**Quality of life:** Studies that assessed qualities of life employed different tools. The EQ-5D and Short-Form Health Surveys (SF-36 and SF-12) were the most popular in these studies. One study reported the effect of smartphone-based CR intervention on two components of the EuroQoL: the EQ-5D index and EQ-5D visual analogue scale points [24]. Meta-analysis of the two studies did not show a significant improvement in the EQ-5D index (MD: 0.04; 95% CI –0.01–0,08, p = 0.11). Varnfield et al. reported a significant improvement in the quality of life compared to the control group (p < 0.001) [19]. Five trials investigated the EQ-5D VAS to subjectively measure patients’ quality of life, and the results of the meta-analysis was not statistically significant (MD: 2.93; 95% CI –0.40–6.26, p = 0.08). We did not perform meta-analyses on study reports that utilize the Short Form Health Survey questionnaires because sufficient data was unavailable. Forest plots of the quality of life measured using the EQ-5D index and VAS scores are illustrated in Figure 7.

**Figure 8:**
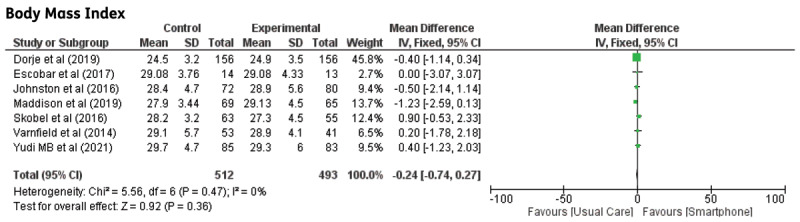
The effect of smartphone-based CR compared to centre-based CR/usual care on Body mass index.

**Body Mass Index (BMI):** Eight studies investigated the differences in body mass index (BMI). There were no significant differences observed (MD: –0.24; 95% CI –0.74–0.27, p = 0.36) The graph for BMI is illustrated in Figure 8.

## Discussion

Current practice guidelines for CR, especially those in phase II, still rely on a face-to-face approach in hospitals or rehabilitation centres. However, dropout rates are relatively high owing to barriers related to distance, transportation, self-motivation, social composition, and support [25]. Furthermore, in the COVID-19 pandemic era, attempts to contain the spread of infection were maximised, while allowing patients to receive optimal care. The potential of telemedicine has been widely recognised and encouraged by the European Society of Cardiology. A previous review reported the benefits of mobile phone interventions for secondary prevention of cardiovascular diseases, including increased adherence to pharmacological treatment, meeting exercise goals, and achieving blood pressure targets [26].

Earlier reviews of telehealth CR have incorporated diverse intervention models. Therefore, the telehealth platforms assessed in this review were narrowed down to smartphone usage. As smartphones become almost ubiquitous in people’s daily lives, they are becoming more confident and interested in using gadgets. Our study assessed the effects of telehealth interventions, specifically smartphone-based CR, on the functional capacity of CHD patients. To the best of our knowledge, this is the first systematic review and meta-analysis to examine the use of smartphones for CR in the CHD population.

The main findings were that smartphone-based CR enhanced functional capacity, as measured by VO_2_ max and a 6-MWT. A significant improvement in smoking cessation rate was also observed. Although our results indicated no significant results for other cardiometabolic risk factors, a favourable outcome was observed in systolic blood pressure. Patients also reported a better quality of life, as measured subjectively using the EQ-5D VAS. There was some evidence of heterogeneity among studies. The characteristics of the different methods and intensities of exercise training were more likely to contribute to variability; therefore, the influence of exercise programs on intervention delivery and effectiveness warrants further consideration.

Despite the remote delivery nature of the interventions and variable exercise programs, most patients reaped a substantial number of benefits in terms of physical tolerance. VO_2_ max has been established as an independent predictor of cardiovascular mortality, all-cause mortality, and cardiovascular risk factors in healthy individuals and CHD patients [27,28]. In a study by Keteyian et al., CVD- and all-cause mortality was reduced by up to 15% with every 1 mL/kg/min increase in VO_2_ max in CHD patients [29]. The 6-MWT test also serves as a prognostic tool in stable CHD patients. A decrease of 104 meters in the 6-MWT group was associated with a 55% increase in the prevalence of cardiovascular events [30]. Interestingly, the included trials that assessed functional capacity in this review had a wide range of follow-up durations, from 6 weeks to 1 year.

We conducted a subgroup analysis to evaluate the impact of different follow-up periods on two parameters: VO2max and 6-MWT. We divided them into two groups, six weeks to six months of follow-up, and six months to 12 months of follow-up. Our findings revealed a statistically significant improvement in VO2max, especially the six-month to 12-month follow-up, while the rest of the group did not yield statistically significant results. In a study by Mohold et al., patients undergoing cardiac rehabilitation were followed up for six months and 30 months, and the results indicated a significant improvement in VO2max during the initial six-month follow-up period for both groups. However, a decline in VO2max was observed in both groups after a 30-month follow up [31]. Conversely, a study done by Pratesi et al. reported a contradicting result, which showed no improvement in functional capacity for a 12-month follow-up period. Notably, the study by Pratesi et al. was done on geriatric patients aged 75 years and above [32]. Currently, a comprehensive summary of long-term benefits of cardiac rehabilitation remains elusive, and no study has yet provided a comprehensive overview of this subject matter.

Despite the varying lengths and frequencies of the interventions, a minimal level of heterogeneity in these outcomes was detected. The trial by Varnfield et al. [19] was performed for only six weeks, which might explain why statistical significance was not achieved, despite significant improvements within each group. One-year follow-up data provided sustainable benefits, as outlined in the study performed by Lunde et al [12]. Regardless, the optimal duration, intensity, and frequency of these interventions remain unknown. It is possible that with a longer intervention duration, adherence and completion rates would diminish as motivation levels decline. This assumption is supported by the centre-based CR performed in a study by Bock et al. A significant change in the perceived benefits of exercise was not achieved in CHD patients undergoing exercise rehabilitation after 3 months [33]. Additionally, Maddison et al. also provided similar evidence. CHD patients showed favourable changes in exercise-related motivation at the 12-week assessment, such as task self-efficacy, barrier self-efficacy, and confidence in adhering to CR programs performed remotely or at the centre. However, these results declined as the CR program extended beyond 12 weeks to 24 weeks. These results require further supporting evidence from a study on the dose-dependent relationships with adherence, especially involving the use of smartphone-based interventions.

There is no doubt that there is an imperative need to improve the cardiovascular risk profiles of CHD patients, especially after an acute attack, to alleviate the burden of subsequent cardiovascular events. A 10 mmHg reduction in systolic blood pressure resulted in a lower risk of major CVD events by 20%, stroke by 27%, heart failure by 28%, and all-cause mortality by 13% in a meta-analysis involving over 610,000 adults, including those with CHD [34]. Of note, our study review did not demonstrate an overwhelming benefit in cardiovascular risk factors, such as blood pressure and lipid profiles. These outcomes were likely confounded by the secondary prevention pharmacotherapy and patient adherence. Furthermore, these risk factors were reasonably controlled upon entering the CR program.

A high level of heterogeneity was detected among studies that evaluated systolic blood pressure for >24 weeks. When the trials by Dorje et al. [13] and Skobel et al. were removed, the heterogeneity became null and the effect size remained insignificant. The study participants in the trial by Dorje et al. were larger than those in other studies. The reported outcome of systolic blood pressure in the study by Skobel et al. was based on a small sample size in the intervention group that could not have produced a significant effect size [21]. Our results are contradictory to reviews assessed by Clark et al. and Neubeck et al., whereby total cholesterol and systolic blood pressure improved significantly after telehealth CR. These reviews included studies that assessed medium-to long-term outcomes [35,36]. Moreover, the trials included in our review were conducted in developed countries, where there is generally a higher level of knowledge and awareness about CVD. In our study, BMI also showed null heterogeneity, and there were no differences in BMI between the smartphone and usual care groups in any of the included studies. These findings contradict those of other telehealth studies by Marquez et al., who found that telehealth intervention was effective and could improve BMI and glucose levels [37]. Another study from Ahmed et al. found that BMI was significantly improved after the cardiac rehabilitation on CHD patients [38].

The mean age of the subjects in these studies ranges from 54–63 (see Appendix 4). Despite the old age population, no study mentions any difficulties in the usage of smartphones. This might be the result of these studies’ inclusion criteria, which require all subjects to have sufficient smartphone literacy. Older adults are known to face more challenges adapting to mobile health applications compared to younger ones due to limited perceptual, motor, and cognitive capabilities, particularly in visual and auditory capacity, hand-eye coordination, and information processing capacity [39]. Studies have shown that the older population is still slow and inconsistent in adopting and using mobile health [40,41,42]. Therefore, it is crucial to comprehend the factors that can either enable or hinder the acceptance of mobile health among older adults [43].

Health literacy plays a pivotal role in healthcare delivery and outcomes. It is associated with limited knowledge of health conditions and medications, increased healthcare costs, and poor prognosis in terms of mortality and morbidities [44]. Community awareness of the dangers of CVD remains lacking in developing countries. The dynamics of secondary preventive efforts are highly influenced by the double burden of pre-transitional (malnutrition and communicable diseases) and post-transitional (non-communicable diseases, such as stroke, CVD, and cancer) diseases. In contrast to the shift towards industrial markets observed in developed countries, the public’s receptiveness to advocating lifestyle behavioural changes regarding CVD is lower [45]. Therefore, extrapolating these study results to a low- to middle-income country requires care and adaptation to the needs of each community’s sociocultural milieu.

Moreover, the included trials employed several methods of delivery available through smartphones, such as smartphone software-enabled systems connected to wearable devices or mobile applications alone or in combination with telephone calls and automated text messages. Feedback and monitoring via telephone calls could provide additional benefits to patients in terms of motivational support. This evidence is supported by a meta-analysis that separately assessed telephone support and remote monitoring in post-discharged heart failure patients. Telemonitoring was effective in reducing mortality and heart failure-related hospitalisations, whereas telephone support was effective only in reducing heart failure-related hospitalisations [46]. Furthermore, diverse components of interventions are also evident in this review. Most studies provided comprehensive feedback on exercise training, education on cardiovascular diseases, associated modifiable behavioural risk factors, dietary habits, and psychosocial support, whereas some provided minimal to no feedback. Each component has unique advantages; therefore, identifying them separately yields more conclusive results. Despite the varying intervention components, it is important to remember that brief interventions that encourage autonomy and choice have a longer-lasting impact. With that in mind, this review provided evidence that with such comprehensive modules of secondary prevention programs, the cessation of smoking habits was successfully achieved. Patients who quit smoking after their first CVD event were less likely to experience recurrent major adverse cardiovascular event or all-cause mortality [47]. Therefore, smoking cessation should be promoted as key educational material.

As demonstrated by Shi et al., patient education for secondary prevention can also reduce symptoms of anxiety and depression in CHD patients, which are associated with psychological well-being [48]. Evaluation and recognition of psychological well-being and quality of life are crucial components of CR. Our study presented inconclusive results regarding the superiority of smartphone-based CR in improving the patients’ quality of life based on the EQ-5D index. The number of studies evaluating changes in quality of life based on the EQ-5D index is relatively minimal. The EQ-5D index can estimate quality-adjusted life years (QALY), which are used to analyse incremental cost-effectiveness in the economic evaluation of an intervention [49]. We acknowledge that patients perceived a better quality of life, as represented by the higher EQ-5D VAS score, which can be explained by the improvement in their functional capacity compared with baseline measurements. This is an important observation because it indicates that CR interventions via smartphones do not negatively impact a patient’s quality of life.

Because various questionnaires were used in other studies, they were not included in the meta-analysis to avoid heterogeneity. Although some of these survey tools display resemblance in some domains of the questions, they cannot be used interchangeably; for example, the EQ-5D and SF-12. The EQ-5D questionnaire is the most widely used, and its utility scores can be used to calculate the cost-utility ratio in an economic analysis based on QALY. Although the SF-12 can be transformed into six-dimensional health state classification (SF-6D) scores for cost-effectiveness analysis purposes, the SF-6D seemed to produce better results in patients with worse EQ-5D outcomes and vice versa [50]. Additionally, patients who underwent PCI or coronary artery bypass graft surgery were likely to report optimal health on the EQ-5D, but not on the SF-6D, compared to patients with acute myocardial infarction. Hence, the differences in outcomes could be affected by patient characteristics and disease severity. Therefore, standardised mean differences were not evaluated. Further studies investigating the health economics of smartphone-based CR and secondary prevention are required.

Some limitations highlighted in our study include the variety of CR models prescribed to patients in terms of duration, frequency, length, intensity, and the variety of tools for outcome measurements. Standardised exercise-based CR protocols fine-tune these outcomes. These studies also underrepresented women. Men have a higher propensity to develop coronary heart disease at a younger age. However, a slow demographic shift was observed in CVD patients. CVD is still a major cause of death in women over the age of 65 years [51]. Therefore, further studies extending participation to the older population are needed to increase the generalisability of the current study outcome. Despite the preconceived notion of limited technology use in older patients, there have been reports that mobile device application engagement is comparable to that of younger patients, particularly with user-friendly and thoughtful personalised smartphone apps [52,53].

Despite these limitations, the quality of the trials included in this review was mostly good, with low levels of bias. The narrow telehealth platform evaluated in this review also contributes to the body of evidence suggesting that smartphone-based CR can be relied upon with confidence for the delivery of secondary prevention strategies. With the specific niche of the study population reviewed in our study, such as those with CHDs, smartphones are a solid method for delivering interventions with desirable outcomes in these patients.

Smartphone-based interventions are relatively inexpensive, accessible anytime at the patient’s own comfort, and easy to use. Clearly, the choice to participate in secondary prevention programs should always reflect patient preferences, anticipation, risk profiles, funding, and access to healthcare. The use of such interventions should also involve careful monitoring and motivational feedback from the healthcare team to maintain meaningful relationships with patients. The development of the most optimised version of this technology is highly competitive and is an integral part of the future of healthcare.

## Conclusion

Smartphone-based CR improves functional capacity (VO_2_ Max and 6-MWT) in CHD patients, as measured by VO_2_ max and 6-MWT. A positive impact was also observed on patients’ smoking habits. However, we did not find evidence that smartphone-based CR can influence lipid profile, blood pressure, HbA1c levels, BMI, or quality of life. Owing to the variability in the methods of exercise/physical training employed, further research involving a more standardised CR protocol needs to be considered. Smartphone technology has the potential to transform cardiovascular care with far-reaching effects.

## Additional File

The additional file for this article can be found as follows:

10.5334/gh.1253.s1 Study Tables. Appendices 1 to 5.
