# Supplementary material for: Smartphone-Based Cardiac Rehabilitation Program Improves Functional Capacity in Coronary Heart Disease Patients: A Systematic Review and Meta-Analysis
Source: Glob Heart. 2023 Aug 10;18(1):42. doi: 10.5334/gh.1253 (PMC10417941; doi:10.5334/gh.1253)
Supplement: Study Tables. — Appendices 1 to 5. [file gh-18-1-1253-s1.pdf]

Smartphone-based Cardiac Rehabilitation Program Improves Functional Capacity in Coronary Heart Disease Patients: A Systematic Review and Meta-Analysis

STUDY TABLES

Appendix 1. Keywords for search terms

Database	Keywords*	No of hits
PubMed	(((((((((((coronary heart disease[MeSH Terms]) OR (angina[MeSH Terms])) OR (ischemic heart disease[MeSH Terms])) OR (acute coronary syndrome[MeSH Terms])) OR (acute myocardial infarct[MeSH Terms])) OR (coronary heart disease)) OR (angina)) OR (ischemic heart disease)) OR (acute coronary syndrome)) OR (acute myocardial infarct)) AND (((((((maximal oxygen consumption[MeSH Terms]) OR (six minute walking test[MeSH Terms])) OR (maximal oxygen consumption)) OR (peak VO2 max)) OR (six minute walking test)) OR (exercise capacity)) OR (functional capacity))) AND ((((((((smartphone[MeSH Terms]) OR (online mentoring[MeSH Terms])) OR (video recording[MeSH Terms])) OR (text message[MeSH Terms])) OR (smartphone)) OR (online mentoring)) OR (video recording)) OR (text message))	25
Medline	((Coronary heart disease/ OR Acute coronary syndrome/ OR Angina Pectoris/ OR Ischemic heart disease/ OR Myocardial infarct/) AND (smartphone OR Smartphone/ OR Internet/ OR telemedicine/ OR Cell Phone/ OR Software/ OR text message OR text message/ OR cell phone/ OR video recording/ OR video call OR Education, distance/ OR Mentoring/ or online mentoring) AND (Cardiac rehabilitation/ OR cardiac rehabilitation OR exercise rehabilitation OR Exercise Therapy/ OR Secondary Prevention/ OR secondary prevention) AND (Exercise tolerance/ OR exercise capacity OR Oxygen consumption/ OR Physical Endurance/ OR maximal oxygen consumption/ OR Peak MET OR six minute walking test)	34
Embase	((Coronary AND heart AND disease) OR (Acute AND coronary AND syndrome) OR (Angina AND Pectoris) OR Ischemic heart disease/ OR (Myocardial AND infarct)) AND ((smartphone OR Internet OR telemedicine OR (Cell AND Phone) OR (video AND call) OR (online AND mentoring)) AND ((Cardiac AND rehabilitation) OR (exercise AND rehabilitation) OR (Secondary AND Prevention)) AND ((Exercise AND capacity) OR maximal oxygen uptake/ OR Endurance OR (Peak AND metabolic AND equivalent AND of energy) OR six minute walk test/)	48

Cochrane	(Coronary heart disease OR acute coronary syndrome OR acute myocardial infarct) AND (smartphone OR application OR online coaching OR telemedicine) AND (Cardiac rehabilitation OR exercise rehabilitation OR secondary prevention) AND (Exercise tolerance OR functional capacity OR maximal oxygen consumption OR exercise)	41
Manual Search		3
Total hits		151

*MeSH term is indicated as slash (/) after keyword

Appendix 2. PRISMA Flow diagram to illustrate study selection process

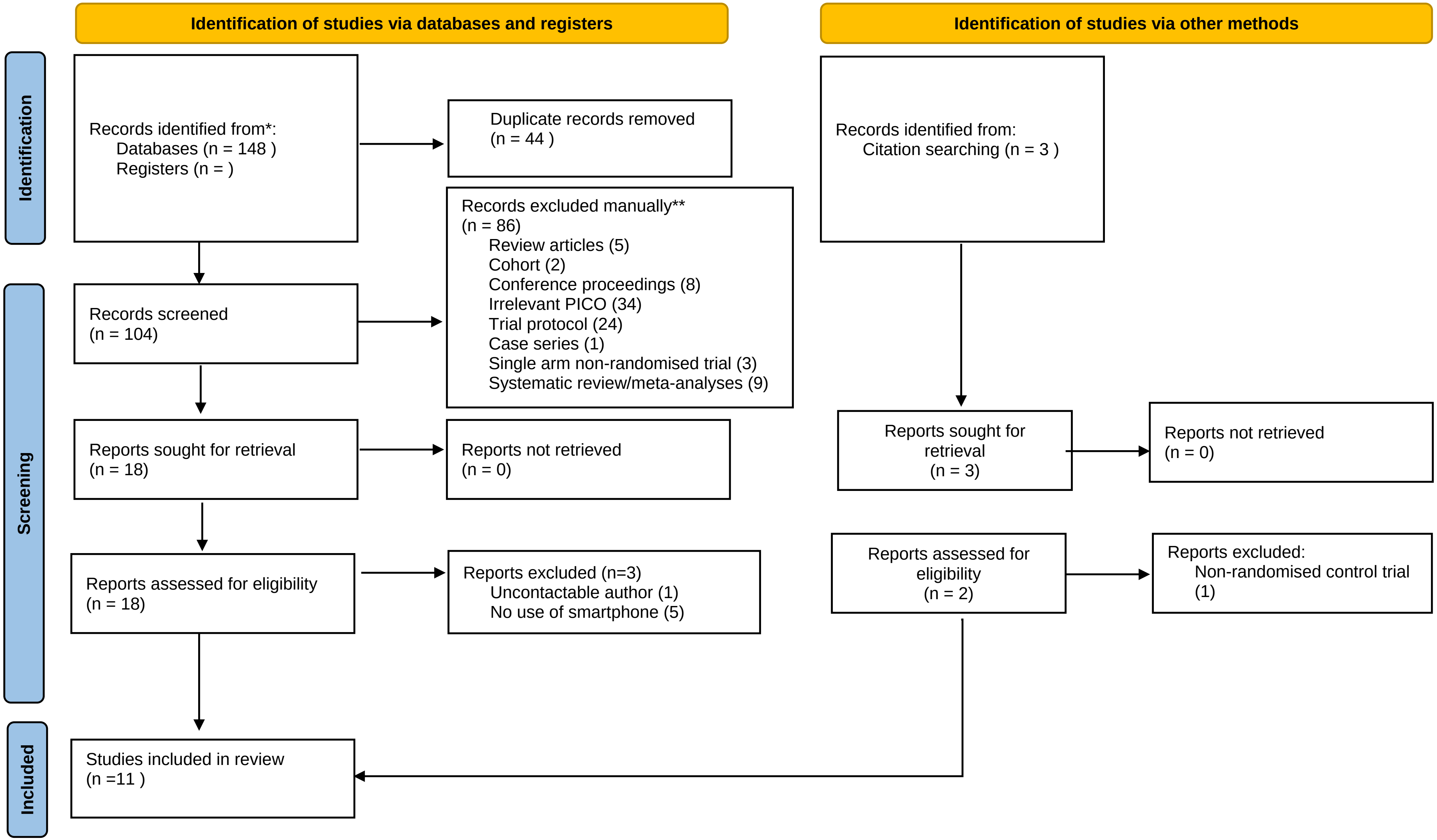

Appendix 3. Summary of study characteristics

No	First Author, Year	Study Origin	Population inclusion criteria	Sample size		Intervention		Comparator	Follow-up duration	Primary outcome Intervention vs Control group Mean change (46)	Secondary outcome Intervention vs Control group Mean change (46)
				IG	CG	Smartphone-based delivery method	Key components of intervention				
1	Yudi MB, 2021(23)	Melbourne, Australia	Patients >18 years with ACS and documented CHD on angiography (coronary artery stenosis >50%, treated either medically or PCI, own a smartphone	83	85	Smartphone application to track activity (linked to accelerometer)	Exercise prescription, dynamic tracking of CVD RF, dietary habits, heart health education, education on secondary prevention pharmacotherapy, interactive and personalised feedback and support	Usual care	8 weeks	6MWT<br>116.6 (75) vs. 91.4 (109.7), P = 0.02<br>*Signficant*	**SBP**: -1.2 (1.30) vs -1.2 (1.39);p=0.8<br>**DBP**: 8.4 (20.72) vs 10.1 (22.99);p=0.79<br>**TC** : -1.4 (1.48) vs -1.41(1.58);p=0.67<br>**LDL-c**: 0.000 (-0.407, 0.407); p=0.8<br>**HbA1C** : -0.2 (1.66) vs -0.1 (1.35);p=0.38<br>**Smoking cessation rate**: 13/25 (52%) vs 11/24 (45.8%)
2	Lunde P , 2020(12)	Norway	Patients age >40 years<br>Owner and user of an Android or Apple smartphone<br>Able to read and understand Norwegian or English	42	41	App-based on smartphone	Goal and tasks added to the app, with automatic reminders of tasks and individualised motivational feedback	Usual care	1 year	Peak VO2<br>1.5 (3.2) vs -1.0(3.3)	**SBP**: 8.6(16.4) vs 7.8(21.3)<br>**DBP**: 5.6 (11.4) vs 2.8 (10.3)<br>**TC** : 0.0 (1.0) vs -0.1 (0.5)<br>LDL-c : 0.1(0.9) vs 0.0 (0.4)<br>**EQ-5D VAS**: 7.9 (16.7) vs 0.5 (13.8)
3	Dorje T, 2019(13)	Shanghai, China	Patients > 18 years with documented CHD (including MI and unstable or stable angina) who were treated with PCI during	156	156	WeChat platform on smartphone	Education about cardiovascular health and disease, physical activity prescription, healthy nutritional advice,	Standard care. WeChat was used for sending follow-up	2 months and 6 months	**6MWT**<br>2 months :<br>49.9(120.4) vs 32.8(119.6));p=0.038<br>6 months :<br>54.2(120.15) vs	**SBP**<br>8 weeks: -2.9(18.45) vs 1.6 (15.04);p=0.45<br>24 weeks: -2.2 (18.38) vs 8.3 (20.93);p=0.029

			admission, own smartphone, active WeChat account/willing to create one, sufficient Chinese language proficiency to enable communication with the CR and secondary prevention coach via WeChat				and support for medication adherence, psychological wellbeing, and modification of coronary heart disease risk factors	visit reminders.		38.5(111.20);p=0.037	**TC**<br>8 weeks: -0.4 (1.442) vs (-0.3 (1.28);p=0.12<br>24 weeks: -0.3 (1.562) vs -0.3 vs(1.220);p=0.71<br>48 weeks: -0.4(1.389) vs 0.0 (1.280); p=0.042<br><br>**LDL-c**<br>8 weeks: -0.1(1.220) vs 0.0 (1.280);p=0.26<br>24 weeks: -0.2(1.220) vs -0.1 (1.0);p=0.22<br>48 weeks:-0.2(1.166) vs 0.1 (1.063); p=0.042<br><br>**Smoking cessation rate**<br>8 weeks: 126/134(94%) vs 117/131(89%)<br>24 weeks: 102/116 (88%) vs 120/131 (92%)
4	Skobel E, 2016(21)	German, British and Spanish	Presence of CHD after acute MI or elective coronary intervention, EF ≥30 %, willing to exercise,<br>eligible for CR and ability to use computer and Internet.	19	42	Smartphone-based guided training	Individual training performance was closely monitored and exercise prescriptions were continuously reviewed and adjusted as needed. This was done by a dedicated team of sport physicians and exercise scientists, Patients in the IG	Report on daily physical activities on a paper dairy.	6 months	**Peak VO2**<br>1.76 ± 4.1 vs −0.4 ± 2.7; p = 0.005	**SBP**: 6 (16) vs −8 (12); p =0.003<br>**DBP**: 1.8 (9) vs −5 ± 9; p=0.01<br>**LDL-c**: −2.4 (26.2) vs −10.5 (64);p = 0.57<br>**EQ-5D VAS**: 0.64 ± 13.9 vs 0.54 ± 10.7; p=0.98

							were evaluated with respect to usability of the system, knowledge about heart-related health, exercise habits and adaption efforts.				
5	Escobar R, 2017(22)	Malaga, Spain	Patients with stable ischaemic cardiomyopathy who had undergone revascularisation by either stent-angioplasty or by-pass surgery at moderate risk<br>Aged ≤ 75, with a good cognitive level, a capacity to perform aerobic exercise on a treadmill or stationary bike, and knowledge of how to use a smartphone or tablet were included in this study.<br>At least one of the following inclusion criteria: ventricular dysfunction using ejection fraction (EF) 40–55%, functional capacity 5–7 METS, and/or raised blood	14	13	NUUBO remote ECG monitoring devices with application pre-installed in smartphone	Exercise at home (walking program for 1h at 70% of reserve HR for at least >=2 days a week<br>Go the cardiac centre once a week for a supervised physical exercise identical to CG	Centre-based CR 3x/week (24 sessions) and encouraged to exercise at home	2 months	**METS**<br>0.73 (3.812) vs 1.35 (2.608);p=0.49	**SBP**: 1.0 (29.27) vs 3.47(31.39) ;p=0.8<br>**DBP**: 0.64 (14.68) vs 3.07(16.63);p0.62<br>**TC** : -0.175 (0.973) vs 0.1999 (1.06);p=0.1<br>**LDL-c**: -0.237 (0.7856) vs 0.1842(0.7378); p=0.06<br>**HbA1c**: 0.1 (1.80) vs 0.13 (1.671);p=0.9

			pressure with exertion.								
6	Fang J, 2019(15)	Shantou, China	Low risk after PCI, living with at least one other person, ability to accept, receive and send mobile phone messages.	33	34	Telephone call weekly<br>Remote monitoring system (belt strap + sensor, application installed on smartphone, computer servers and a web portal)<br>Same CHD booklet as control<br>2 home visits during a 6-week interval	Instructed to outdoor walking/jogging CHD booklet	Standard post-PCI care protocol (paper-based and self-study CHD booklet, biweekly outpatient review)	6 weeks	**6MWT**<br>48.2 (45.99) vs 34.77 (50.13) ;p=0.006	**SBP**: -1.03 (23.83) vs -1.82 (23.19); p=0.139<br>**DBP**: 0.0 (18.66) vs -1.09(16.84); p=0.552
7	Maddison R , 2019(20)		Clinically stable, English-speaking adults >18 years with CHD within 6 months (atherosclerosis, angina pectoris, MI, coronary revascularisation)	68	72	Smartphone, chest-worn wearable sensor, bespok[illegible] smartphone and web ap[illegible] and custom middleware. Sensor provides information on heart an[illegible] respiratory rates, single [illegible] ECG and accelerometer.<br>SMS for education on behavioural change	Individualised exercise prescription, exercise monitoring and coaching + theory-based behavioural strategies to promote exercise and habitual physical activity	Centre-based CR	12, 24 weeks	**Peak VO2**<br>3.3(12.46) vs 1.69 (9.56)	**SBP**<br>12 weeks: -3.9 (22.76) vs -3.4 (24.07)<br>24 weeks: -1.7 (24.34) vs -3.6(25.14)<br><br>**DBP**<br>12 weeks : -1.72(14.85) vs -2.86(14.11)<br>24 weeks : -2.07 (14.82) vs -1.72(15.09)<br><br>**EQ-5D index**<br>12 weeks: 0.02 (0.134) vs -0.01(0.164)<br>24 weeks: 0.01 (0.134) vs -0.02(0.164)

8	Song Y, 2020(14)	Beijing, China	(1) Age≤ 75 years old; (2) Diagnosed as stable CHD by coronary angiography; (3) without physical or mental disorders affecting exercise; (4) Skillful in using software such as WeChat and telemonitoring software.	48	48	Telemonitoring software installed in smartphone with wearable devices (heart rate belts). communication with medical staffs via WeChat and telephone call	Exercise training, feedback by patients on exercise status (frequency, intensity, and time)	Standard treatment + routine clinical follow-up within 6 months after enrolment No in-hospital CR	6 months	**Peak VO2**<br>1.89 (6.62) vs 0.24 (6.652)	
9	Widmer RJ , 2017(18)		Patients treated with PCI for ACS, willingness to participate, access to internet	37	34	Online and smartphone-based CR program (apps) + standard CR	Dietary and exercise habits, educational materials on healthy lifestyles	Standard CR	3 months	CV-related ED visits and rehospitalizations	**SBP**: 0 (13.3) vs -2.1 (16.5); p=0.67<br>**DBP**: 4.0 (11.9) vs -4.4 (13.1);p=0.93<br>**TC** *:* -0.887 (1.00) vs -1.39(1.342) ;p=0.24<br>**LDL***:* -0.584 (0.874) vs -1.135 (0.825);p= 0.26<br>**HbA1c**: -0.7 ± 2.0 vs -0.8 ± 1.1;p= 0.98<br>**Peak VO2**: 1.1 ± 2.9 vs 0.6 ± 1.1;p=0.67
10	Varnfield, 2014(19)	Melbourne, Australia	Post-MI patients referred to CR	53	41	Text message and preinstalled audio and video files<br>Smartphone preinstalled with health diary and activity monitoring app, BP monitor, and weight scale<br>Weekly phone call	Health and exercise monitoring, delivery of motivational and educational materials	Centre-based CR	6 weeks, 6 months	**6MWT**<br>60 (111.04) vs 47(131.14);p=0.4	**SBP**:-2.8 (23.77) vs 0.4 (20.790);p=0.4<br>**DBP**: -2.5 (12.44) vs 1.4(12.16) ;p=0.03<br>**TC :** -0.26 (1.414) vs -0.63 (1.308);p=0.2<br>**LDL**: -0.26 (1.414) vs -0.63 (1.308);p=0.5<br>**EQ-5D Index** : 0.08 (0.214) vs -0.01 (0.258); p=0.01

11	Johnston, 2016(17)	Uppsala, Sweden	1) Females or males aged >18 years<br>2) Diagnosed with STEMI/NSTEMI with treatment initiated in-hospital and prior to randomisation with ticagrelor 90 mg twice daily and for the duration of 1 year according to guidelines.<br>3) having daily access and knowledge how to handle a personal smartphone<br>4) Swedish language skills<br>5) Willingness and ability to participate in scheduled follow-up visits	86	80	Web-based application installed on their own smartphones	Extended drug adherence e-diary-, exercise-, weight-, and smoking- modules	A simplified tool containing only a simplified drug adherence e-diary without the secondary prevention educational modules installed on their own smartphones	2 visits:<br>6-10 weeks post discharge<br>6 months (study end)	Medication adherence	**SBP**: -0.6 (14.55) vs -1.1 (17.83)<br>**LDL-c**-: -1.8 (0.672) vs -1.0 (0.666)<br>**EQ-5D VAS**:14.9 (22.51) vs 9.1(24.78)<br>**Smoking cessation rate:** 16/22(72.7%) vs 5/12 (41.67%)

Appendix 4. Patient characteristics in included studies

Study design	Trial	Age (Mean SD)		Gender (Male/Female in %)		Diabetes		Hypertension		Smoker		Dyslipidemia	
		**Intervention**	**Control**	**Intervention**	**Control**	**Intervention**	**Control**	**Intervention**	**Control**	**Intervention**	**Control**	**Intervention**	**Control**
RCT	Yudi MB et al (2019)	56.8(9.9)	56.2(10.2)	86.5/13.5	83.3/16.7	21.7%	15.3%	47.0%	43.5%	30.1%	28.2%	48.2%	48.2%
RCT	Lunde P et al (2020)	59.5(9.1)	58.4(8.2)	84.2/15.8	71.4/28.6	NR	NR	NR	NR	4.8%		NR	NR
RCT	Dorje et al (2019)	61.9 (8.7)	59.1(9.4)	81/19	82/18	NR	NR	NR	NR	56.0%	57.0%	NR	NR
RCT	Skobel et al (2016)	60 (50,65)	58 (52, 67)	91/9	87/13	NR	NR	NR	NR	NR	NR	NR	NR
RCT	Escobar et al (2017)	56.5(6.01)	55.64(11.35)	100/0	100/0	42.9%	7.1%	64.3%	78.6%	14.3%	14.3%	50.0%	35.7%
RCT	Fang et al (2018)	60.2 (9.4)	61.4 (10.2)	63.6/36.4	61.8/38.2	27.3%	28.2%	45.5%	41.2%	NR	NR	NR	NR
RCT	Maddison et al (2020)	61(13.2)	61,5(12.2)	84.2/15.8	87.5/12.5	80.0%	82.0%	79.9%	82.0%	83.3%	0.0%	80.0%	82.0%
RCT	Song Y et al (2020)	54.17 (8.76)	54.83 (9.13)	89.6/10.4	83.33/16.67	54.2%	41.7%	60.4%	77.1%	66.7%	54.2%	77.1%	64.6%
RCT	Widmer et al (2017)	62.5 (10.7)	63.6 (10.9)	85/15	78/22	32.0%	13.0%	82.0%	70.0%	3.0%	15.0%	97.0%	93.0%
RCT	Varnfield et al (2014)	54.9(9.6)	56.2(10.1)	83/17	91/9	15.0%	20.0%	55.0%	44.0%	15.0%	10.0%	55.0%	46.0%
RCT	Johnston N et al (2016)	56.8(8.0)	58.4(8.6)	82.6/17.4	78.8/21.2	9.3%	16.3%	46.5%	47.5%	25.6%	15.0%	27.9%	16.3%

Appendix 5. Risk of bias in included trials

Rias of bias domains

	D1	D2	D3	D4	D5	Overall
Yudi MB et al (2021)	+	+	+	+	+	+
Lunde P et al (2020)	+	+	+	×	+	−
Dorje et al (2019)	+	+	+	+	+	+
Skobel et al (2016)	+	+	×	+	+	−
Escobar et al (2017)	+	+	+	+	+	+
Fang et al (2018)	+	−	×	×	−	×
Maddison et al (2020)	+	+	+	+	+	+
Song Y et al (2020)	+	+	+	+	+	+
Widmer et al (2017)	+	+	+	+	+	+
Varnfield et al (2014)	+	+	+	×	+	−
Johnston N et al (2016)	+	−	−	×	+	×

Domains:

D1: Bias arising from randomization process

D2: Bias due to deviations from intended intervention

D3: Bias due to missing outcome data

D4: Bias in measurement of the outcome

D5: Bias in selection of the reported result

Judgement

× High

− Some concerns

+ Low
